# Prediction of mobilized hematopoietic stem cell yield in patients with multiple myeloma: Usefulness of whole-body MRI-derived indices

**DOI:** 10.1371/journal.pone.0283241

**Published:** 2023-03-31

**Authors:** Miyuki Takasu, Ryo Higashino, Takahiro Sueoka, Saki Kawai, Nobuko Tanitame, Akihisa Tamura, Makoto Iida, Takakazu Kawase, Tatsuo Ichinohe, Kazuo Awai

**Affiliations:** 1 Department of Diagnostic Radiology, Hiroshima City Hiroshima Citizens Hospital, Hiroshima, Japan; 2 Department of Diagnostic Radiology, Hiroshima Prefectural Hospital, Hiroshima, Japan; 3 Department of Immune Regenerative Medicine, International Center for Cell and Gene Therapy, Research Promotion and Support Headquarters, Fujita Health University, Aichi, Japan; 4 Department of Hematology and Oncology, Research Institute for Radiation Biology and Medicine (RIRBM), Hiroshima University, Hiroshima, Japan; 5 Department of Diagnostic Radiology, Graduate School of Biomedical Sciences, Hiroshima University, Hiroshima, Japan; University of Missouri, UNITED STATES

## Abstract

**Introduction:**

High-dose chemotherapy followed by autologous stem cell transplant is the mainstay of treatment for multiple myeloma (MM). The purpose of this study was to evaluate the ability of MRI-derived indices to predict mobilized hematopoietic stem cell yield.

**Materials and methods:**

In this exploratory pilot work, we retrospectively analyzed 38 mobilization procedures for MM. Successful mobilization procedure was defined as a total yield of >4.0×10^6^ CD34^+^ cells/kg. Univariate and multivariate analyses were performed to identify factors with a significant effect on successful mobilization from among clinical characteristics including number of prior lines of therapy, period from diagnosis to harvest, type of monoclonal protein (M protein); and radiological characteristics including total diffusion volume (tDV), median apparent diffusion coefficient (ADC) of tDV, and mean fat fraction of bone marrow calculated by MRI.

**Results:**

Univariate analyses showed that relatively poor mobilization was significantly associated with M protein of Bence-Jones type and with median ADC of tDV (P = 0.02 and P = 0.004, respectively). Multivariate analyses using these two indices showed that median ADC of tDV was a significant predictive factor for adequate mobilization (P = 0.01), with an area under the curve of 0.784 (cutoff value, 1.18×10^−3^ mm^2^/s; sensitivity, 72.7%; specificity, 87.5%).

**Conclusion:**

The present data indicate that median ADC of tDV is a predictive factor for relatively poor mobilization of hematopoietic stem cells in MM patients undergoing autologous stem cell transplant.

## Introduction

Autologous hematopoietic stem cell transplantation (HSCT) after high-dose chemotherapy is a common and effective treatment for multiple myeloma (MM) patients [1]. HSCT requires the prior collection and cryopreservation of hematopoietic stem cells (HSCs) for successful engraftment [2]. HSCs are obtained from peripheral blood (PB) following administration of granulocyte colony-stimulating factor (G-CSF) [3]. However, many patients are poor mobilizers and fail to obtain sufficient numbers of HSCs for successful autologous HSCT [4, 5]. Lower yield of PB-CD34^+^ cells is related to an increased risk of prolonged cytopenia and morbidity following high-dose chemotherapy [6]. Thus, it would be useful to predict those at potential risk of poor response prior to the mobilization procedure, particularly given the toxicity of chemomobilization and the significant financial burden.

Previous reports have identified many factors predictive of poor mobilization with G-CSF, including patient age, previous chemotherapy, melphalan treatment, lenalidomide treatment, multiple chemotherapies, incomplete disease remission, white blood cell count, blood hemoglobin level, and baseline platelet count [7–11].

Diffusion-weighted imaging (DWI) is one of the MRI sequences shown to be useful in imaging of bone marrow (BM). DWI is sensitive to the mobility of free water molecules, which can be quantified as the apparent diffusion coefficient (ADC) calculated from multiple image acquisitions at varying b values (typically 0 and 1000 s/mm^2^). On diffusion-weighted images, areas of high cellularity (limited movement of water molecules) have bright signal intensity and low ADC values, and those of low cellularity (increased movements of water molecules) have low signal intensity and high ADC values. Tumors that have limited mobility of free water include hematologic malignancies, such as MM and lymphoma [12, 13]. A significant correlation between ADC and BM infiltration by plasma cells has also been demonstrated [14], which enables quantitative assessment of disease burden [15] and response to chemotherapy [16]. To the best of our knowledge, there are no previous reports on the relationship between total yield of PB-CD34^+^ cells and indices calculated from MRI. Direct detection of CD34^+^ cells using MRI prior to PB stem cell harvesting would be ideal for predicting total CD34^+^ count. However, this is impossible in the clinical setting, because the cells must form a tissue of some size to be detectable by MRI.

Given these capabilities of MRI for characterization of BM infiltration of myeloma cells, there is clinical interest in evaluating whether DWI can be used as an adjunct tool in the premobilization work-up of patients with MM. The purpose of this study was to validate the predictive ability of MRI-derived indices for mobilization failure.

## Materials and methods

### Patient selection

This retrospective, single-institution study was approved by the Institutional Review Board of Hiroshima University Hospital, with a waiver for informed consent.

In this exploratory and a pilot study, we utilized data on consecutive patients with MM who underwent autologous HSCT, whole-body MRI including both whole-body DWI and mDixon Quant sequence of lumbar BM prior to HSCT at Hiroshima University Hospital between 2015 and 2020. The International Myeloma Working Group diagnostic criteria were used [17].

### Definition of poor mobilization

The number of CD34^+^ cells reported as sufficient to provide a high probability of successful HSCT ranges from 2 × 10^6^ to ≥6 × 10^6^ CD34^+^ cells/kg [6, 18, 19]; however, there is no consensus regarding the total CD34^+^ count indicative of poor mobilization. Therefore, the present study utilized an operational definition that classified patients with a total yield of <4.0×10^6^ CD34^+^ cells/kg as relatively poor mobilizers.

### MRI and semi-automated workflows

Whole-body MRI examinations were performed with a 3-T system (Ingenia; Philips Healthcare, Best, The Netherlands) using head, anterior torso array, and integrated posterior coils. Patients were scanned in the supine position at 4 stations, to cover from the vertex to the knees. The imaging parameters are summarized in the S1 Table and are available at protocols.io (dx.doi.org/10.17504/protocols.io.bavbie2n). A whole-body coronal 3D-spoiled gradient echo pulse sequence (mDixon Quant) was performed with six evenly spaced echoes.

All images were visually assessed independently by two readers (M.T., 26 years of experience in spinal imaging, Reader 1; and T.K., 23 years of experience in hematology, Reader 2) to ascertain image quality for data processing regarding artifact-related degradation including motion and ghosting, with prior knowledge of the clinical information. The authors were blinded to each other’s results and the review was performed after conceptualization of this study.

**Fig 1:**
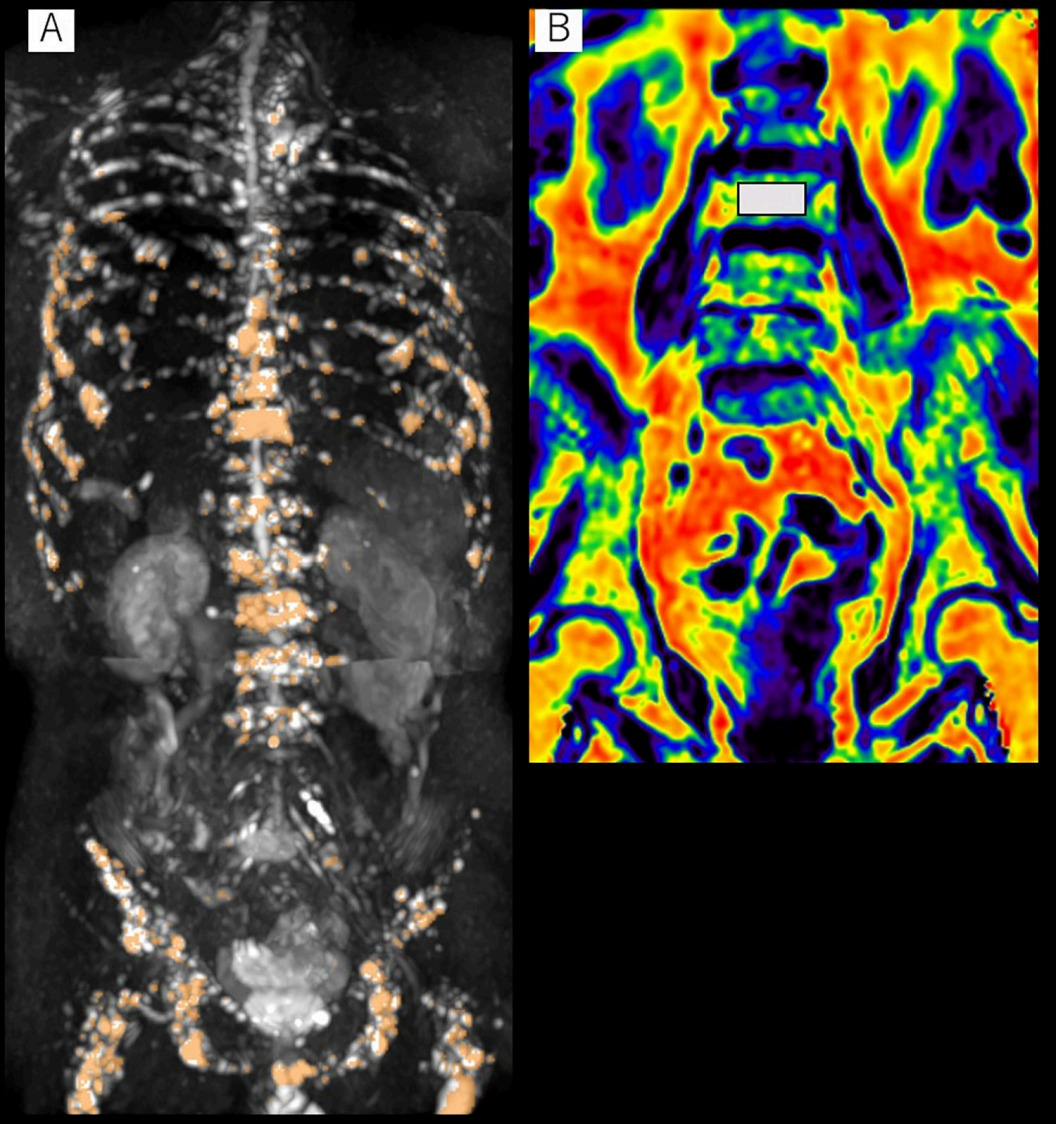
Semi-automated segmentation of myeloma lesions and measurement of fat fraction on MR images. (A) Coronal maximum intensity projection image acquired by diffusion-weighted imaging with a computed b-value of 1000 s/mm^2^. For estimation of whole-body volumetric tumor burden by measurement of apparent diffusion coefficient (ADC), voxels showing an ADC between 0.55 ×10^−3^ and 2.0 ×10^−3^ mm^2^/s are extracted (indicated in orange) as an estimate of total diffusion volume, as a surrogate marker for tumor burden. (B) Coronal fat fraction map obtained by modified Dixon Quant sequence. The region of interest (white rectangle) for measuring fat fraction is placed on the L3 vertebral body on a midcoronal image. The region of interest cannot be placed from L1 to L3 on a single image because the mid-coronal level of each vertebral body is different due to lumbar lordosis.

For whole-body DWI, image processing was performed by Reader 1 using medical imaging software (BD score; PixSpace, Japan). Semi-automated segmentation of myeloma lesions was performed in the following steps (Fig 1):

1. On a maximum intensity projection (MIP) DWI image (b value = 1000 s/mm^2^), the contrast in signal between disease and background tissues was maximized on visual inspection. A single optimal value of 97 was then determined as the background threshold for all studies to provide an initial classification of disease from background.
2. After removal of the background, images were converted into binary images with two gray levels using a gray-level threshold value, which was automatically determined by the Otsu discriminant analysis method [20]. White and black regions corresponded to disease and to space other than disease, respectively.
3. On an MIP and multi-planar reformat viewer, areas of high signal other than the skeletal system (e.g., brain, lymph node, intestine) were manually removed by the author. To eliminate the influence of T2 shine-through effect, voxels showing an ADC ≤2.0 ×10^−3^ mm^2^/s were extracted from the remaining high-signal areas (Fig 1A).

As an estimate of total diffusion volume (tDV), voxels with an ADC above the threshold of 0.55 ×10^−3^ mm^2^/s (as determined in a previous study) were extracted from the remaining areas of high signal, for each patient [16]. Median ADC for all voxels in the tDV was then calculated.

Water-only, in-phase, opposed-phase, and fat-only images acquired with whole-body coronal 3D mDixon Quant sequence were used to evaluate fat fraction (FF) in the BM. The FF map was generated from the ratio of the signal intensity in the fat-only image divided by the signal intensity in the in-phase image (Fig 1B). The rectangular regions of interest on a midcoronal image of the FF map of the L1–L3 vertebral bodies were used to calculate mean FF because they are less affected by degenerative disc disease. The range in area of the regions of interest placed for BM was 270–485 mm^2^.

### Clinical data

We reviewed patients’ demographic and clinical characteristics, blood data, and medical history, as follows: sex, age, number of prior therapies, number of cycles of induction chemotherapy, specific myeloma drugs; white blood cell, neutrophil, and platelet count, hemoglobin level; and response to induction therapy before mobilization, time between diagnosis and PB stem cell harvesting, type of monoclonal protein, and type of G-CSF.

### Primary endpoint

The primary endpoint was to determine variables that enable prediction of relatively poor mobilizers of hematopoietic stem cells. Moreover, the predictive abilities of clinical and MRI-derived indices for relatively poor mobilization were compared.

### Statistical analyses

Only patients for whom a complete dataset was available were included in this study.

Readers 1 and 2 reviewed the clinical and laboratory data from patients’ medical record, including treatment received, disease assessments, and the results of standard laboratory evaluations by the same two authors who assessed the image quality of MRI.

Data are presented as the mean ± standard deviation or median (range), and categorical variables are presented as the number and percentage.

Patient characteristics, biochemistry results, and MRI-derived indices before mobilization were compared using the Mann–Whitney U test. Variables showing values of *P* <0.05 in univariate analysis were included in the multivariate analysis.

Simple regression analysis was used to examine correlations between each independent variable and the total number of PB-CD34^+^ cells. The predictive equation to yield the total number of PB-CD34^+^ cells was determined using multiple regression with stepwise forward and backward inclusion of independent variables. Receiver-operating characteristic (ROC) curves were plotted for predictive variables to assess performance for predicting relatively poor mobilization.

Probability values were considered significant for values of *P* <0.05. All analyses were performed with a spreadsheet application (Office Excel version 3.20; Microsoft).

## Results

### Patient demographics

We identified 38 patients with MM who received autologous HSCT with G-CSF during the study period. There were 15 male patients (mean age, 60.0 years; age range, 44–72 years) and 23 female patients (mean age, 60.9 years; age range, 36–70 years). Of the overall patients, 33 had symptomatic myeloma and 5 had solitary plasmacytoma. Chromosome 17p deletion was detected in one patient and translocation t (14;16) was present in one patient.

### Treatment regimens and transplant procedures

Of the 38 patients, 28 had been newly diagnosed with MM and the remaining 10 patients had received up to 6 prior chemotherapy regimens (median, 2 regimens) and up to 2 radiotherapy regimens.

The induction chemotherapy regimens were bortezomib + dexamethasone (n = 13), bortezomib + cyclophosphamide + dexamethasone (n = 10), bortezomib + lenalidomide + dexamethasone (n = 7), lenalidomide + dexamethasone (n = 4), daratumumab + lenalidomide + dexamethasone (n = 2), and pomalidomide + dexamethasone (n = 2).

The HSCT procedures were performed under the guidelines of the Japanese Society of Hematology, the Japan Society for Hematopoietic Cell Transplantation, and the Japanese Society of Myeloma. Briefly, all patients were mobilized with cyclophosphamide (2000 mg/m^2^ total) over 2 days followed by G-CSF (filgrastim at 400 μg/m^2^ (n = 27); lenograstim at 5 μg/kg (n = 5); lenograstim + plerixafor at 0.24mg/kg (n = 5); filgrastim + plerixafor at 0.24mg/kg (n = 1)), subcutaneously once a day for 4 days.

S2 Table lists the clinical information (including the regimen of induction therapy and type of M protein) for six patients who were given plerixafor.

### Patient demographics and clinical characteristics

Table 1 lists the patient demographics, biochemistry findings, and clinical characteristics according to the response to HSC mobilization. All seven patients with Bence-Jones protein were relatively poor mobilizers (*P* < 0.05). No significant difference was identified between mobilizers and relatively poor mobilizers for any other variable. However, there was a tendency for more than one line of previous chemotherapy and previous alkylating and lenalidomide therapy to be associated with relatively poor mobilization. Plerixafor was used in six patients but was not related to achievement of adequate mobilization.

**Table 1:** Patient characteristics and biochemistry results.

	All patients	Adequate harvest	Relatively poor mobilization	*P*
PB-CD34^+^ (×10^6^ cells/kg, median, interquartile range)	3.55 (1.43–5.33)	6.05 (4.73–7.30)	1.48 (1.09–2.93)	N/A
Number of patients, n	38	16	22	
Age (years; median, range)	63.00 (36–72)	63.5 (36–66)	63.0 (52–72)	0.90
Sex: male/female, n (%)	15 (39)/23 (61)	7 (18)/9 (24)	8 (21)/14 (37)	0.65
Laboratory data				
Type of monoclonal protein				0.02
Ig G, n (%)	22 (58)	13 (34)	9 (24)	
Ig A, n (%)	8 (21)	3 (8)	5 (13)	
Ig D, n (%)	1 (3)	0 (0)	1 (3)	
Bence-Jones κ/λ, n (%)	7 (18)	0 (0)	7 (18)	
R-ISS stage at diagnosis				0.69
I, n (%)	8 (21)	4 (11)	4 (11)	
II, n (%)	21 (55)	8 (21)	13 (34)	
III, n (%)	4 (11)	1 (3)	3 (8)	
Solitary plasmacytoma, n (%)	5 (13)	3 (8)	2 (5)	
Clinical status at mobilization				0.66
Complete remission, n (%)	6 (16)	2 (5)	4 (11)	
Very good partial response, n (%)	8 (21)	5 (13)	3 (8)	
Partial response, n (%)	20 (53)	8 (21)	12 (32)	
Stable disease, n (%)	3 (8)	1 (3)	2 (5)	
Progressive disease, n (%)	1 (3)	0 (0)	1 (3)	
Diagnosis–mobilization period (months; median, range)	7 (3–63)	5 (4–25)	7 (3–63)	0.27
<12 months, n (%)	30 (79)	13 (34)	17 (45)	0.77
≥12 months, n (%)	8 (21)	3 (8)	5 (13)	
Previous radiotherapy, n (%)	9 (23)	2 (5)	7 (18)	0.17
Number of cycles of induction chemotherapy (n, median, range)	4 (2–10)	4 (3–10)	4 (2–9)	0.46
No. of lines of previous chemotherapy (median, range)	1 (2–7)	1 (2–7)	2 (1–5)	0.006
1 line, n (%)	22 (58)	12 (32)	10 (26)	0.07
≥2 lines, n (%)	16 (42)	4 (11)	12 (32)	
Specific myeloma drugs				
Previous alkylating and lenalidomide therapy, n (%)	8 (21)	1 (3)	7 (18)	0.06
Previous alkylating therapy, n (%)	10 (26)	7 (18)	3 (8)	0.09
Previous lenalidomide therapy, n (%)	9 (24)	6 (16)	3 (8)	0.19
Growth factor				0.37
Filgrastim, n (%)	27 (71)	13 (34)	14 (37)	
Lenograstim, n (%)	5 (13)	2 (5)	3 (8)	
G-CSF and plerixafor, n (%)	6 (16)	1 (3)	5 (13)	
Blood test				
White blood cell (10^3^/μL)	2.81 ± 2.48	2.28 ± 2.32	3.19 ± 2.58	0.18
Neutrophil (10^3^/μL)	1.84 ± 1.88	1.50 ± 1.76	2.08 ± 1.97	0.37
Platelet (10^4^/μL)	156 ± 86	128 ± 84	176 ± 83	0.09
Hemoglobin (g/dl)	11.1 ± 1.7	11.1 ± 1.6	11.1 ± 1.8	0.74

Note. Values of blood tests are presented as the mean ± standard deviation or median (range).

Abbreviations: PB, peripheral blood; N/A, not applicable; R-ISS, revised International Staging System; G-CSF, granulocyte colony-stimulating factor.

### MRI variables

All MRI examinations were performed before or shortly after initiation of remission induction chemotherapy. The median time interval between MRI and harvest was 4.3 ± 1.9 months.

The two readers considered all acquired images to be suitable for processing to obtain MRI indices.

Table 2 presents the MRI-derived indices according to patient response to mobilization. Median lesion ADC was significantly higher in relatively poor mobilizers than in those who achieved adequate stem cell yield. There was no significant difference between mobilizers and relatively poor mobilizers in terms of tDV of myeloma lesions or FF in lumbar BM.

**Table 2:** Comparison of MRI-derived indices according to their response to mobilization.

	Adequate harvest	Relatively poor mobilization	*P*
Time interval between MRI and harvest (months)	4.3 ± 1.4	5.1 ± 2.1	0.14
MRI data			
Whole-body DWI			
Total diffusion volume (ml)	95.3 ± 81.0	57.8 ± 59.1	0.36
Median ADC (×10^−3^ mm^2^/s)	0.96 ± 0.24	1.24 ± 0.26	0.004
mDixon Quant			
Fat fraction (%)	34.3 ± 23.1	45.6 ± 25.6	0.25

Note. Values represent mean ± standard deviation.

DWI, Diffusion-weighted imaging; ADC, apparent diffusion coefficient.

### Predictive ability of clinical and MRI variables for relatively poor mobilization

Results of the regression analyses are displayed in Table 3. Median ADC and Bence-Jones myeloma were both significantly correlated with relatively poor mobilization (P = 0.01, P = 0.04, respectively).

**Table 3:** Predictive factors significantly associated with relatively poor mobilization by multivariate analysis.

Predictive factor	Dichotomization	*β ± standard error	95% confidence interval	P
Median ADC	N/A	0.674 ± 0.260	0.15, -1.20	0.01
Type of monoclonal protein	Bence-Jones κ/λ or not	-0.379 ± 0.179	-0.74, -0.02	0.04

Note. Values represent mean ± standard deviation.

Abbreviations: ADC, apparent diffusion coefficient; N/A, not applicable.

**Fig 2:**
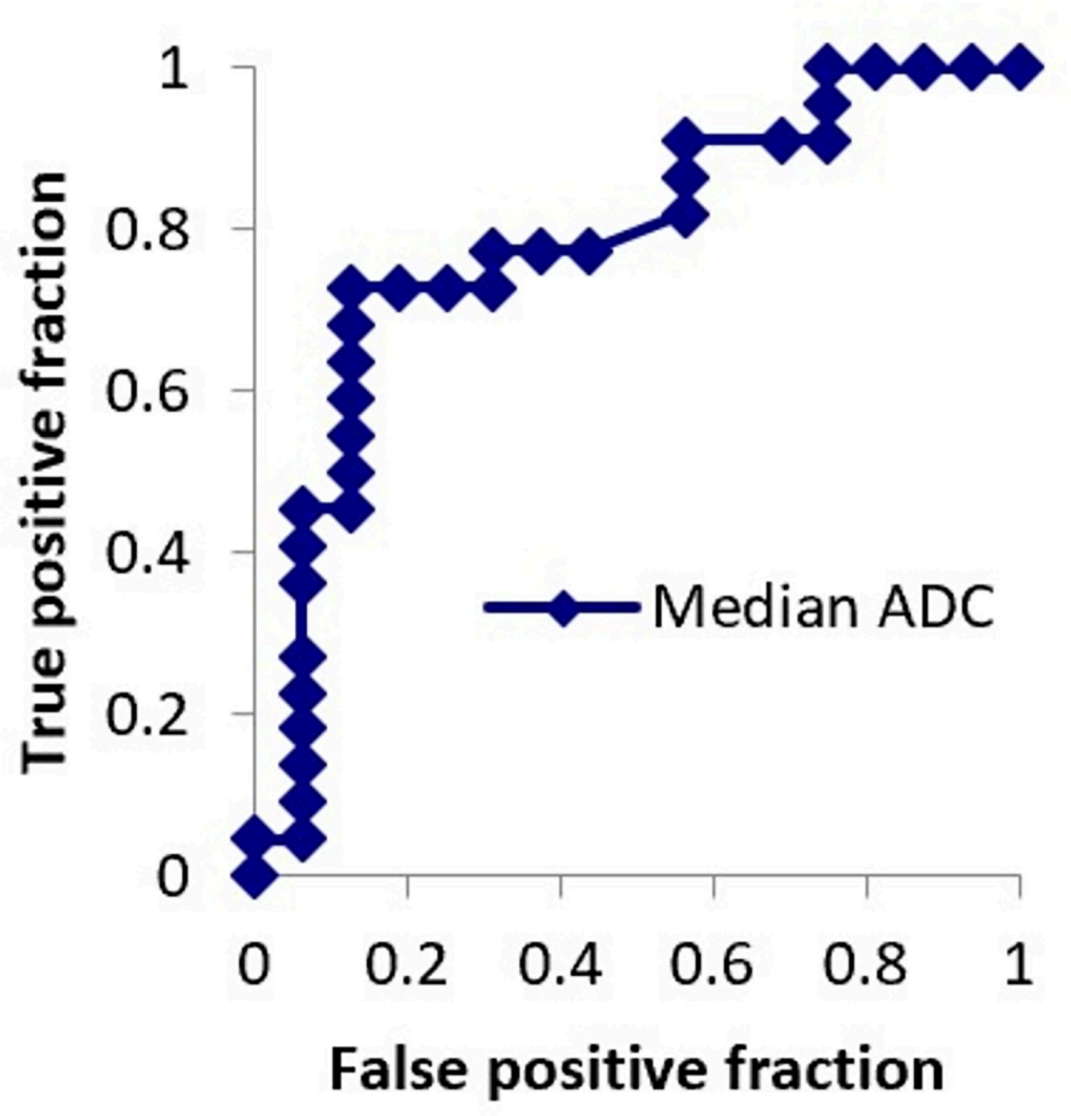
Receiver-operating characteristic (ROC) curve for median apparent diffusion coefficient. The area under the ROC curve is 0.784.

The ROC curve for median ADC is shown in Fig 2. Area under the ROC curve was 0.784. A cutoff threshold of > 1.18 × 10^−3^ mm^2^/s enabled prediction of relatively poor mobilization with 72.7% sensitivity and 87.5% specificity.

**Fig 3:**
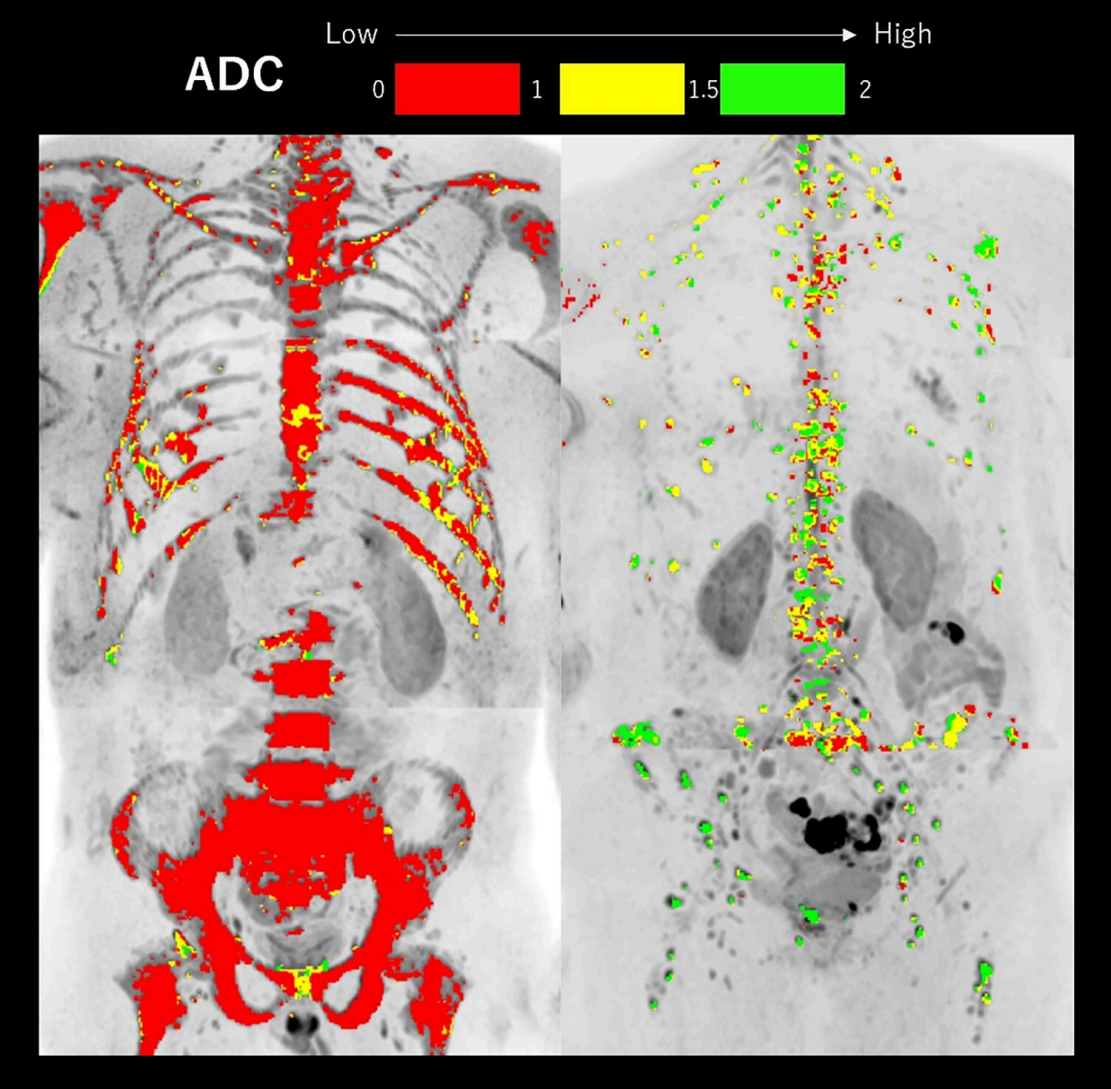
Representative whole-body diffusion-weighted imaging (DWI) for the patient classifications of mobilizer and relatively poor mobilizer. Left image, mobilizer: Whole-body DWI of a 47-year-old man with symptomatic myeloma who had an adequate number of collected CD34^+^ cells (4.4×10^6^ CD34^+^ cells/kg). Median ADC of the total diffusion volume was 0.78×10^−3^ mm^2^/s. Right image, relatively poor mobilizer: Whole-body DWI of a 59-year-old woman with symptomatic myeloma who had an inadequate number of collected CD34^+^ cells (3.8×10^6^ CD34^+^ cells/kg). Median ADC of the total diffusion volume was 1.53×10^−3^ mm^2^/s.

Representative images of a mobilizer and a relatively poor mobilizer are shown in Fig 3.

## Discussion

This study evaluated the ability of MRI-derived indices and demographic and clinical parameters to predict PB-CD34^+^ cell yield for autologous HSCT in MM. We found that median ADC in tDV values were significantly higher in relatively poor mobilizers than in patients who achieved adequate stem cell yield. This novel finding suggests that whole-body DWI can be used as a predictive factor for yield of hematopoietic stem cell mobilization.

Factors previously reported to be associated with poor mobilization in MM included age [9], bone marrow involvement with the disease [21], PB Hb level [10], platelet count at time of mobilization, white blood cell count [7], circulating immature cells and baseline platelet count [22], type of G-CSF utilized [9], prior lenalidomide therapy [23, 24], prior melphalan therapy [8, 25], number of prior lines of therapy [8], and extensive irradiation to bone marrow sites [21, 26]. Some of these factors are considered to be related in some way to the mass of hematopoietic BM. For example, hematopoietic bone marrow decreases with age in both sexes [27], and blood cell count reflects the function and volume of hematopoietic BM. Irradiation of BM causes adipocyte infiltration of the BM and subsequent decrease in hematopoietic BM [28].

The present study found no significant difference in tDV between relatively poor mobilizers and those who achieved adequate mobilization. Total diffusion volume is the total number of voxels with an ADC above a certain threshold and can be utilized as a surrogate marker of tumor mass. Therefore, the statistical insignificance of tDV for predicting poor mobilization might not be explained simply as the residual amount of normal hematopoietic BM that is spared from myeloma infiltration.

ADC is a quantitative measure of Brownian motion. Low ADC values indicate microenvironments with high cellular density, whereas high ADC values are found in hypocellular areas where water diffusion is more free. Intracellular water diffusion is more restricted than extracellular water diffusion due to structures such as cell organelles and the cytoskeleton; indeed, it has been estimated that the rate of extracellular water diffusion is two to three times higher than that of intracellular water [29]. The ADC value calculated from DWI is believed to have a basic association with the density of the molecules diffusing in each subcompartment as well as the number of molecules present [30]. We therefore believe that the present finding of a significant correlation between median ADC and relatively poor mobilization might be related to a difference in the fraction of the extracellular water compartment, in which water protons in myeloma lesions reside.

The relationship of percentage of extracellular space to the cellular biology of tumors is controversial. The volume of extravascular extracellular space per unit volume of tissue (V_e_) and the volume transfer constant (K_trans_) indicating the transfer rate can both be quantified as hemodynamic features of dynamic contrast-enhanced MRI based on pharmacokinetic models.

Some authors have reported significantly higher K_trans_ and V_e_ values in histological grade 1–2 tumors than in grade 3 tumors for endometrial carcinoma [31], and V_e_ in malignant breast lesions was significantly lower compared with normal glands [32], which suggest a tendency of aggressive tumors to have hypercellularity and reduced extracellular space. Conversely, Satta et al. [31] have demonstrated that high-risk endometrial carcinoma showed reduced intratumor perfusion, probably due to inadequate neoangiogenesis, which leads to tissue hypoxia and necrosis, resulting in liquefied areas in the tumor (i.e., extracellular space). Myeloma bone lesions are usually surrounded by fat-dominant BM, in contrast to solid tumors that are usually in the vicinity of normal soft tissue and vasculature. Therefore, we speculate that following induction therapy, the BM in relatively poor mobilizers has less growth medium for normal hematopoietic BM compared with that of mobilizers, because of the larger fraction of extracellular space due to intratumor necrosis and the lower possibility of angiogenesis from surrounding fat-dominant BM in these patients. However, dynamic contrast-enhanced MRI was not performed in the present patients to evaluate extravascular extracellular space in focal lesions of myeloma because many had impaired renal function [33].

In the context of the BM condition after induction chemotherapy, MRI immediately before a mobilization procedure would indicate the BM environment directly associated with HSC yield. In the present study, we examined the ability of pre-induction MRI to predict mobilization failure, and focused on myeloma tissue biology because the number of collected HSCs does not depend on the drug used, with new drugs being approved continuously.

Because BM is composed of hematopoietic marrow and fat marrow, FF in BM is inversely correlated with the degree of BM cellularity [34]. Fat fraction calculated in MRI is a noninvasive means of quantifying fat within the BM, liver, and musculoskeletal system, and is reported to be useful in the prediction of remission status after chemotherapy for MM [16], in osteoporosis [35], in differentiation of acute benign and neoplastic compression fractures of the spine [36], in evaluation of hepatic steatosis [37], in prediction of severity of rotator cuff tear [38], and in evaluation of severity of cancer cachexia by psoas muscle fat infiltration [39]. The present study found no significant difference between mobilizers and relatively poor mobilizers in terms of FF in the lumbar BM. The water fraction (i.e., 1 –FF) is the fraction of proton signal other than fatty marrow in the BM and its origin includes both normal hematopoietic BM and tumor cells. Therefore, the absolute value of FF might not show significant correlation with BM condition.

More than one line of previous chemotherapy and previous alkylating plus lenalidomide therapy showed a tendency toward poor mobilization, although the difference did not achieve statistical significance. C-X-C chemokine receptor type 4 surface expression [40] and downregulation of transcription factor PU.1 by lenalidomide [41] have been reported as underlying molecular mechanisms and their description is beyond the scope of this article.

Our study had several limitations. First, it was performed at a single institution and our sample size was small, which limits statistical power and generalizability. Further study with a larger sample size and definitive data sets is warranted to confirm our results. Second, plerixafor was used for enhancement of hematopoietic stem cell mobilization for 6 of 38 patients in this study. Although no significant difference was demonstrated between mobilizers and relatively poor mobilizers regarding the use of plerixafor, different results might be obtained if its use becomes more universal. Third, there was no single treatment protocol; however, chemotherapy regimens were not significantly biased between mobilizers and relatively poor mobilizers. We therefore did not consider this factor to affect the results. Finally, the readers of MRI were not blinded to the clinical information of the patients or the concept of this study. The cases in this study were selected consecutively according to the selection criteria and the quality of all images was judged by two readers to be acceptable for analysis. Therefore, we believe that this limitation is not significant.

In conclusion, the present findings indicate that median ADC of tDV is a predictive factor for relatively poor mobilizers of HSCs in MM patients receiving autologous stem cell transplant. The non-invasive nature of MRI and the ability to perform whole-body imaging make MRI worthy of consideration for use in pretreatment workup. Based on these promising results, further prospective studies with a larger number of patients are warranted to further evaluate the predictive abilities of MRI.

## Supporting information

S1 Table MRI sequence protocol. (DOCX)

S2 Table Clinical information for six cases who plerixafor was given. (DOCX)

## Abbreviations

HSCT: hematopoietic stem cell transplantation
MM: multiple myeloma; HSCs, hematopoietic stem cells
PB: peripheral blood; G-CSF, granulocyte colony-stimulating factor
DWI: diffusion-weighted imaging; BM, bone marrow
ADC: apparent diffusion coefficient; MIP, maximum intensity projection
tDV: total diffusion volume
FF: fat fraction; ROC, receiver-operating characteristic
V_e_: volume of extravascular extracellular space per unit volume of tissue
K_trans_: volume transfer constant
