# Supplementary material for: Prediction of mobilized hematopoietic stem cell yield in patients with multiple myeloma: Usefulness of whole-body MRI-derived indices
Source: PLoS One. 2023 Mar 31;18(3):e0283241. doi: 10.1371/journal.pone.0283241 (PMC10065245; doi:10.1371/journal.pone.0283241)
Supplement: S1 Table — (DOCX) [file pone.0283241.s001.docx]

**Supporting information**

**S1 Table. MRI sequence protocol**

| Parameter | Sequence | | | | | |
| --- | --- | --- | --- | --- | --- | --- |
|  | Whole-spine sagittal T1-weighted | Whole-spine sagittal STIR | Whole-body axial T2-weighted | Whole body coronal T1-weighted | Whole-body coronal DWIBS | Whole-body coronal mDixon Quant |
| Sequence type | FSE | STIR | FSE | FSE | STIR | 3D SPGR |
| Time (ms) |  |  |  |  |  |  |
| TR | 404 | 5693 | 1000 | 515 | 5411 | 5.7 |
| TE | 10 | 70 | 70 | 15 | 70 | Six evenly spaced echoes |
| Inversion time | N/A | 200 | N/A | N/A | 250 | N/A |
| Slice thickness (mm) | 4 | 4 | 6 | 5 | 4 | 6 |
| Number of slices per station | 15 | 15 | 40 | 34 | 50 | 64 |
| In-plane pixel size (mm) | 1.4×2.7 | 1.5×2.2 | 1.1×1.5 | 1.7×3.5 | 2.3×3.5 | 2.4×2.4 |
| Bandwidth/pixel (Hz) | 576 | 625 | 359 | 435 | 2535 | 128 |
| Acquisition time (min) | 6 | 6 | 6 | 6 | 8 | 1.3 |
| b Value (s/mm^2^) | N/A | N/A | N/A | N/A | 0 and 1000 | N/A |

Abbreviations: DWIBS, diffusion-weighted whole-body imaging with background body signal suppression; mDixon Quant, modified Dixon Quant; FSE, fast spin-echo; STIR, short tau inversion recovery; EPI, echo-planar imaging: 3D SPGR, 3D spoiled gradient- echo; N/A, not applicable.

(dx.doi.org/10.17504/protocols.io.bavbie2n)
