# Supplementary material for: Prediction of mobilized hematopoietic stem cell yield in patients with multiple myeloma: Usefulness of whole-body MRI-derived indices
Source: PLoS One. 2023 Mar 31;18(3):e0283241. doi: 10.1371/journal.pone.0283241 (PMC10065245; doi:10.1371/journal.pone.0283241)
Supplement: S2 Table — (DOCX) [file pone.0283241.s002.docx]

**Supporting information**

**S2 Table. Clinical information for six cases who plerixafor was given**

| Patient No. | PB-CD34^+^ (×10^6^ cells/kg) | Type of monoclonal protein | Regimen of induction therapy | Cycles of induction therapy, n | Clinical status at mobilization | Growth factor |
| --- | --- | --- | --- | --- | --- | --- |
| 1 | 0.9 | BJP | DRd | 3 | CR | Filgrastim |
| 2 | 0.288 | Ig A | DRd | 3 | CR | Lenograstim |
| 3 | 1.46 | BJP | Bd | 7 | PR | Lenograstim |
| 4 | 1.5 | BJP | Bd | 4 | PR | Lenograstim |
| 5 | 0.62 | Ig G | Bd | 3 | PR | Lenograstim |
| 6 | 4.32 | Ig G | VCD | 4 | PR | Lenograstim |

Abbreviations: PB, peripheral blood; BJP, Bence-Jones protein; DRd, daratumumab + lenalidomide + dexamethasone; Bd, bortezomib + dexamethasone; VCD, bortezomib + cyclophosphamide + dexamethasone.
